# Strangulation injury from indigenous rocking cradle

**DOI:** 10.4103/0974-2700.66543

**Published:** 2010

**Authors:** Abhijeet Saha, Prerna Batra, Anuradha Bansal

**Affiliations:** Department of Pediatrics, Mahatma Gandhi Institute of Medical Sciences, Sevagram, India; 1 Department of Pediatrics, Government Medical College and Hospital, Sector 32 B, Chandigarh, India

**Keywords:** Indigenous rocking cradle, infant, strangulation

## Abstract

Indigenously made rocking cradle is frequently used in rural India. We report strangulation from an indigenously made rocking cradle in an 11-month-old female child. The unique mode of injury and its mechanism have been discussed. Strangulation is an important cause of homicidal and suicidal injury in adults but in children it is usually accidental leading to death due to asphyxia as a result of partial hanging. In western countries, it is the third most common cause of accidental childhood deaths, 17% of them being due to ropes and cords. It ranks fourth amongst the causes of unintentional injury in children less than 1 year of age following roadside accidents, drowning and burns. However, in India, strangulation injury is under reported although indigenous rocking cradles are very commonly used in rural India, and they are even more dangerous than the cribs and adult beds as there are no safety mechanisms therein. We report a case of accidental strangulation following suspension from an indigenously made rocking cradle. The unique mode of injury has prompted us to report this case.

## INTRODUCTION

Strangulation is an important cause of homicidal and suicidal injury in adults but in children it is usually accidental leading to death due to asphyxia as a result of partial hanging.[1] In western countries, it is the third most common cause of accidental childhood deaths, 17% of them being due to ropes and cords.[2,3] It ranks fourth amongst the causes of unintentional injury in children less than 1 year of age following roadside accidents, drowning and burns.[4] However, in India, strangulation injury is under reported although indigenous rocking cradles are very commonly used in rural India, and they are even more dangerous than the cribs and adult beds as there are no safety mechanisms therein.[5] We report a case of accidental strangulation following suspension from an indigenously made rocking cradle. The unique mode of injury has prompted us to report this case.

## CASE REPORT

**Figure 1:**
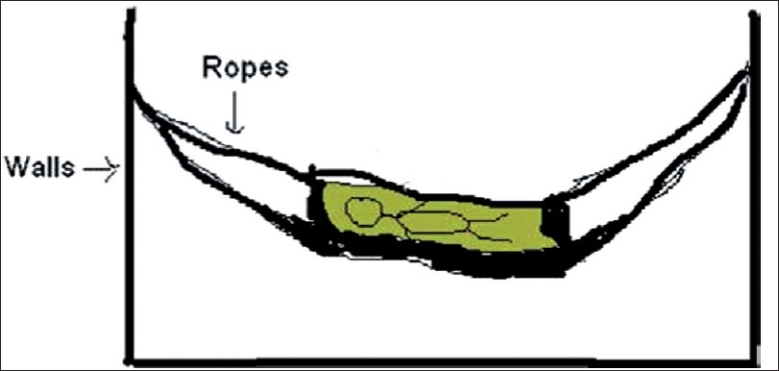
Baby lying in an indigenous rocking cradle

**Figure 2:**
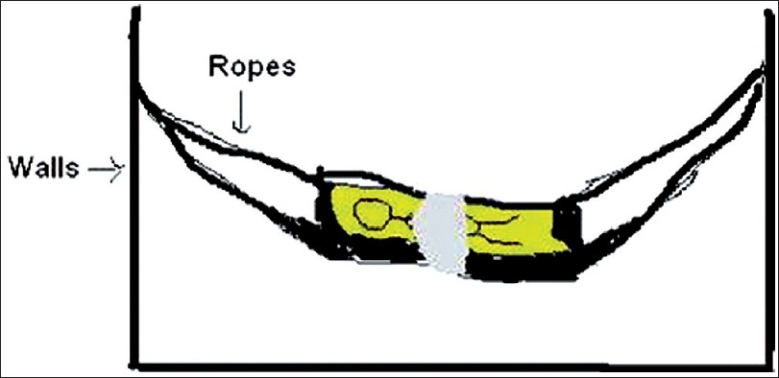
Baby lying tied with a piece of cloth in an indigenous rocking cradle

**Figure 3:**
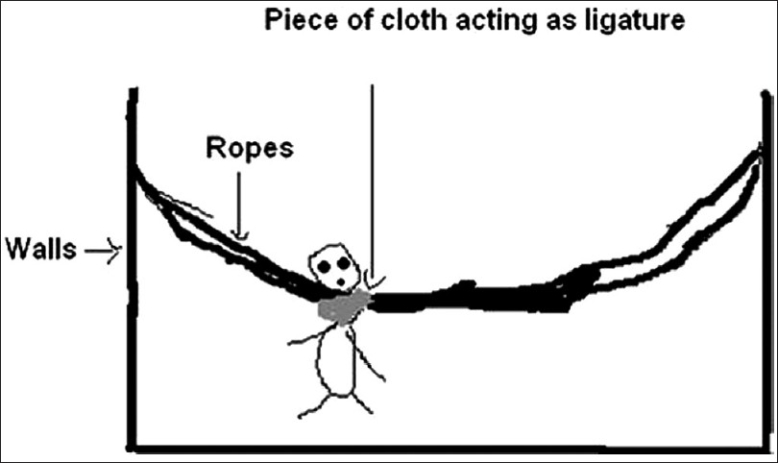
Partial hanging from the rocking cradle

An 11-month-old female child was brought to the casualty services of pediatrics emergency with history of unconsciousness and one episode of generalized tonic-clonic convulsions. According to her mother, the child was sleeping unattended in an indigenously made rocking cradle [Figure 1] while her mother was working in courtyard outside the house. She had tied a piece of cloth over the child’s abdomen to protect the child from falling down [Figure 2]. When the mother came inside, she found the child hanging from the sling. Probably, the unattended child woke up and started struggling in the cradle and the cloth slipped from the abdomen to her neck. She fell down and the cloth got stuck around her neck acting as a ligature to cause partial hanging [Figure 3].

The child was immediately removed from the sling and within 30 minutes she was brought to the hospital. On the way to the hospital, the baby became unconscious and had an episode of generalized tonic-clonic convulsions.

The child was immediately admitted to the emergency room and a formal assessment was done as per the standard protocol. The child was drowsy, had a pulse rate of 110 per minute, respiratory rate of 38 per minute and blood pressure of 102/62 mm Hg. She had peripheral cyanosis and there were a few petechiae over her neck. There was a diffuse ligature mark over the neck along with few petechiae. No other injury mark was noted over the body. There was no evidence of sexual abuse either. Central nervous system examination revealed a Glasgow Coma Scale (GCS) of 12/15, equally reacting pupils with preserved Doll’s eye movements. Muscle tone and deep tendon reflexes were normal with bilateral flexor plantar response. Rest of the systemic examination was normal. She was started on oxygen by hood, maintenance intravenous fluids and started on continuous hemodynamic and neurological monitoring. On admission, her investigations were as follows: hemoglobin 10.5 g/dl, total leukocyte count 11,450/mm^3^, serum sodium 141 mEq/l, serum potassium 4.5 mEq/l and blood glucose 98 mg/dl. Arterial blood gases were normal. Neck X-ray did not reveal any hyoid or cervical vertebral fracture. Rest of the skeletal survey also did not reveal any bony fracture or dislocation. As per the hospital policy, a medico-legal case was registered and the police official-in-charge of that area was informed, who investigated the case and ruled out any foul play.

There was no recurrence of seizure in the hospital, the child was managed symptomatically and she regained consciousness within 6 hours of admission, and hence computerized tomography (CT) scan cranium was deferred. She was discharged after 36 hours with instructions for parents not to leave the child unattended in the cradle/slings. The community health worker did an awareness program in the village on our request.

## DISCUSSION

Accidental strangulation is a potentially fatal injury in children. In a large case series by Feldman and Simss, 8.6% cases of accidental strangulation were reported to be due to clothing entanglement.[6] In a recent study of 28 cases of pediatric and adolescent strangulation from India, 7% of the cases were accidental.[7] Clothing and personal belongings were found to be the most common ligature materials.

Children sleeping unattended in rocking cradles are prone to asphyxiation. Due to the rocking motion of the cradle, the child can slide to the dependent area, his head can be compressed and his nose and mouth may be obstructed leading to suffocation.[8] If the child is lying prone, because of developmental immaturity or mechanical obstruction he may not be able to raise and turn his head to maintain oxygenation, carbon dioxide may increase by rebreathing leading to unconsciousness and death.[8] If the infant has been fed just before returning to the cradle, regurgitated milk and mucus from the nostrils may dampen the bedding which would increase the obstructive effect when compressed against nose and mouth. The struggling infant may fall from the cradle and his neck may be entrapped in the sling used to tie the child in the cradle as it happened in our patient.

This mode of injury has not been reported previously. In this case there was partial hanging, where the weight of a part of the body pulls on the ligature. Here tension applied to neck may occlude the airway leading to asphyxia. This generally occurs above the larynx and below the angle of jaw.[1,2] Neck compression may lead to laryngeal edema.[2,9] Moreover, airway of infants is anatomically vulnerable to obstruction at the level of oropharynx between the soft palate and the skull.[10]

Nearly one-third of cases have seizures due to neurological damage as in the case of our patient. Death in these cases is usually due to hypoxic damage to brain and respiratory system.[11] The exact mechanism of neurological damage and death is not known but hypoxic-ischemic injury seems to be the most likely cause.[9] The following mechanisms have been proposed:[12–14] (1) direct injury to brainstem and spinal cord; (2) mechanical constriction of neck leading to airway obstruction; (3) cardiac arrest due to massive vagal reflexes; (4) jugular venous obstruction; (5) mechanical obstruction to blood flow in carotid arteries.

Spinal cord injuries are uncommon in pediatric strangulation and vagal reflex due to bilateral carotid body stimulation is also unlikely to directly cause brain damage or death.[12] Hence, mechanical constriction of neck seems to be the most likely cause. Airway obstruction occurs as the base of tongue is pushed against the posterior pharyngeal wall and epiglottis folds over the larynx. Obstruction to carotid artery flow is unlikely in children as approximately 5 kg tension on ligature around the neck is required to cause cessation of carotid flow and even then the patency of vertebral arteries is preserved.[1,12] However, only 2 kg tension is required to cause bilateral jugular venous obstruction which may cause cerebral edema and unconsciousness or seizures.[1] This may consequently lead to rise in intracranial tension later in the course of illness, herniation and brain death.

Management of these cases requires intensive monitoring and supportive care, maintenance of airway, circulation and management of seizures and cerebral edema. Bone fractures and laryngeal edema may be associated, thus should be anticipated. Prognosis depends on duration of unconsciousness, presence of seizures, diabetes insipidus or hyperglycemia at admission.[9] The index case had only one of these. Survivors of strangulation injury may have cognitive disabilities later on due to hypoxic ischemic injury to the hippocampus.[15]

## CONCLUSION

To conclude, strangulation injury from indigenously made rocking cradle could be potentially fatal because of its neurological and respiratory complications. Accidental strangulation from these cradles can be avoided if the infants are not left unattended.
